# Rapidly Progressive Neuroendocrine Carcinoma of the Ampulla of Vater Detected During Intraductal Papillary Mucinous Neoplasm Surveillance

**DOI:** 10.7759/cureus.113153

**Published:** 2026-07-22

**Authors:** Takato Inoue, Koichiro Mandai, Ayako Nishimura, Satoru Yasukawa, Takuji Kawamura

**Affiliations:** 1 Department of Gastroenterology, Kyoto Second Red Cross Hospital, Kyoto, JPN; 2 Department of Pathology, Kyoto Second Red Cross Hospital, Kyoto, JPN

**Keywords:** ampulla of the vater, eus-fnb, intraductal papillary mucinous neoplasm (ipmn), neuroendocrine carcinoma(nec), rapidly progressive

## Abstract

A 77-year-old woman undergoing surveillance for an intraductal papillary mucinous neoplasm (IPMN) was found to have new enlargement of the major papilla on esophagogastroduodenoscopy. Contrast-enhanced computed tomography and endoscopic ultrasound (EUS) revealed an ampullary mass with multiple hepatic lesions. Because no tumor exposure was evident on the duodenal mucosal surface, endoscopic ultrasound-guided fine-needle biopsy (EUS-FNB) was performed. Histopathological and immunohistochemical findings, including a high Ki-67 labeling index, confirmed the diagnosis of neuroendocrine carcinoma (NEC). The lesion had not been detected on esophagogastroduodenoscopy 12 months earlier, and magnetic resonance cholangiopancreatography obtained two months before diagnosis had not raised suspicion of ampullary malignancy. Despite biliary drainage and carboplatin-etoposide chemotherapy, the disease progressed rapidly, and the patient died four months after diagnosis. This case highlights the aggressive clinical course of ampullary NEC and the importance of careful evaluation of newly detected ampullary changes during IPMN surveillance. It also demonstrates the practical usefulness of EUS-FNB for establishing a pathological diagnosis in a non-exposed ampullary lesion.

## Introduction

Neuroendocrine neoplasms (NENs) of the gastrointestinal tract and pancreas are classified as well-differentiated neuroendocrine tumors (NETs) and poorly differentiated neuroendocrine carcinomas (NECs) according to the 2019 World Health Organization classification [1]. In contrast to NETs, NECs are high-grade malignancies characterized by marked cellular atypia, high proliferative activity, rapid progression, early metastasis, and poor prognosis [2]. Neuroendocrine neoplasms arising from the ampulla of Vater are rare, and NEC represents an uncommon but highly aggressive subtype [3].

The ampulla of Vater is a clinically important anatomical site because tumors arising in this region frequently cause biliary obstruction and often require multimodal imaging and histopathological evaluation for an accurate diagnosis. However, pathological diagnosis may be challenging when an ampullary lesion is not exposed on the duodenal mucosal surface, limiting the diagnostic yield of conventional forceps biopsy [4,5]. In such situations, endoscopic ultrasound-guided tissue acquisition (EUS-TA) may provide an effective alternative for obtaining adequate tissue for histopathological diagnosis.

Patients with intraductal papillary mucinous neoplasms (IPMNs) undergo periodic imaging surveillance to detect morphological progression of the cystic lesion or the development of pancreatic ductal adenocarcinoma. However, the incidental detection of ampullary NEC during IPMN surveillance has rarely been described.

We report a case of rapidly progressive ampullary small-cell NEC detected during surveillance for branch-duct IPMN. This case highlights the aggressive clinical behavior of ampullary NECs, the importance of careful evaluation of newly detected ampullary abnormalities even during routine surveillance, and the potential role of EUS-guided fine-needle biopsy (EUS-FNB) in establishing a pathological diagnosis in a non-exposed ampullary lesion.

## Case presentation

A 77-year-old woman presented with painless jaundice in June 2024. Her Eastern Cooperative Oncology Group performance status was 0. She had undergone surgery for breast cancer in 1995 and curative endoscopic submucosal dissection (ESD) for early gastric cancer one month before presentation. She had also been diagnosed with multiple branch-duct IPMNs involving the pancreatic body and tail in February 2023. The cysts measured up to 9 mm, without worrisome features, mural nodules, or main pancreatic duct dilatation (main pancreatic duct diameter, 2 mm). She subsequently underwent surveillance every six months without interval changes (Table 1).

**Table 1:** Timeline of the patient's clinical course.

Time point	Clinical course
1995	Surgery for breast cancer
Feb 2023	Multiple branch-duct intraductal papillary mucinous neoplasms (IPMNs) were identified in the pancreatic body and tail (maximum diameter, 9 mm). No mural nodules, main pancreatic duct dilatation (2 mm), or other worrisome features were observed. Surveillance was initiated
Jun 2023	Follow-up esophagogastroduodenoscopy demonstrated no abnormality of the major papilla
Apr 2024	Follow-up abdominal ultrasonography and magnetic resonance cholangiopancreatography showed stable branch-duct IPMNs without interval change. No ampullary lesion, biliary dilatation, or metastatic disease was detected
May 2024	Curative endoscopic submucosal dissection was performed for early gastric cancer
Jun 2024	The patient presented with painless jaundice (Eastern Cooperative Oncology Group (ECOG) performance status 0). Esophagogastroduodenoscopy revealed a newly enlarged major papilla. Contrast-enhanced computed tomography and endoscopic ultrasound demonstrated a 25-mm ampullary tumor with synchronous liver and para-aortic lymph node metastases
Jun 2024	Endoscopic ultrasound-guided fine-needle biopsy established the diagnosis of small-cell neuroendocrine carcinoma. Endoscopic retrograde biliary drainage using a 7-Fr, 5-cm straight plastic stent resulted in prompt resolution of obstructive jaundice
Jul 2024	Chemotherapy with carboplatin and etoposide was initiated
Sep 2024	Computed tomography performed after two cycles of chemotherapy demonstrated progressive disease
Oct 2024	The patient died approximately four months after diagnosis

Follow-up esophagogastroduodenoscopy performed one year before diagnosis showed no abnormality of the major papilla. In addition, abdominal ultrasonography and magnetic resonance cholangiopancreatography performed two months before presentation demonstrated stable branch-duct IPMNs without interval change, biliary dilatation, ampullary abnormalities, or evidence of metastatic disease.

**Figure 1:**
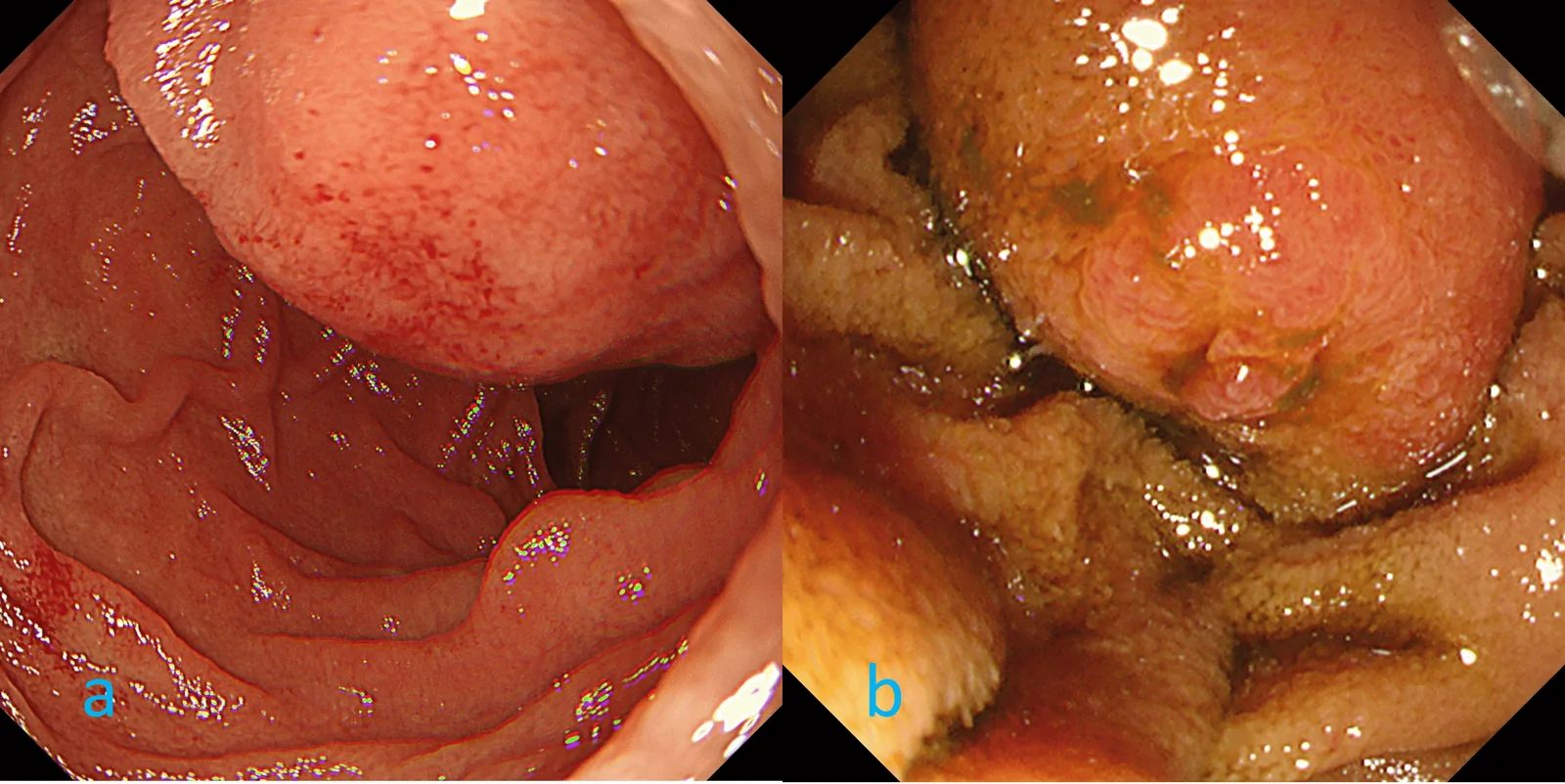
Endoscopic images of the major papilla. (a) Forward-viewing endoscopy revealed an elevated lesion on the oral side of the major papilla. (B) Side-viewing duodenoscopy demonstrated enlargement of the major papilla. No tumor exposure was observed on the mucosal surface.

**Figure 2:**
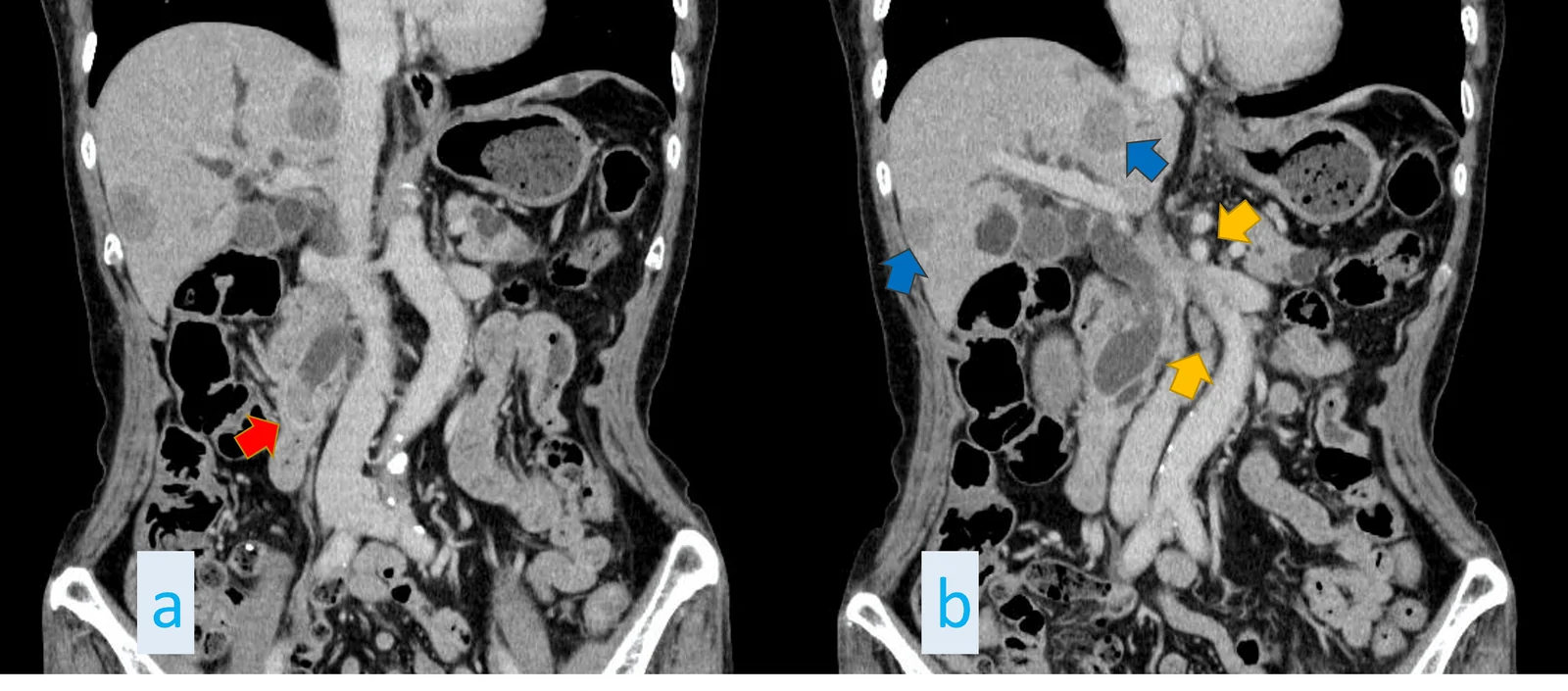
Contrast-enhanced abdominal computed tomography. (a) Contrast-enhanced abdominal computed tomography revealed a 25-mm mass at the ampulla of Vater, showing persistent enhancement from the arterial to equilibrium phases (red arrow), accompanied by dilation of the bile duct and main pancreatic duct. (b) Para-aortic lymph node metastasis (yellow arrow) and multiple hepatic metastases (blue arrows) were observed.

**Figure 3:**
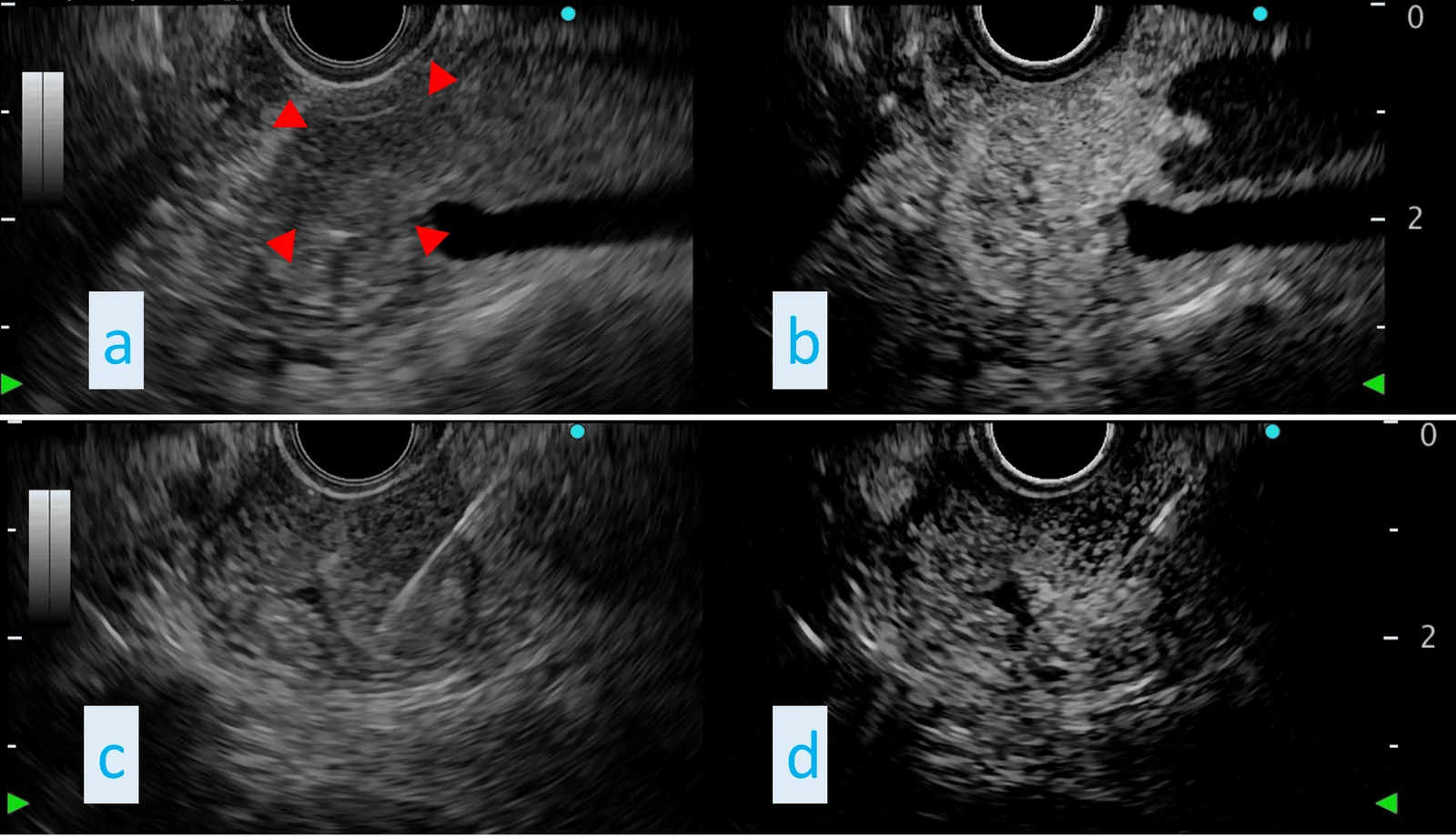
Endoscopic ultrasound (EUS) findings and EUS-guided fine-needle biopsy. (a) Endoscopic ultrasound demonstrated a 25-mm heterogeneously hypoechoic mass at the ampulla of Vater (red arrowheads). Delineation between the mass and the common bile duct was difficult because of intraductal sludge. (b) Contrast-enhanced endoscopic ultrasound using perflubutane demonstrated distinct hyperenhancement of the lesion, allowing clear visualization of the tumor. Dilatation of the common bile duct and main pancreatic duct was also evident. (c, d) Endoscopic ultrasound-guided fine-needle biopsy was successfully performed using a 22-gauge needle without procedure-related complications.

During routine follow-up esophagogastroduodenoscopy after ESD for early gastric cancer, a newly developed elevated lesion was identified at the major papilla (Figure 1). Laboratory tests revealed conjugated hyperbilirubinemia and elevated hepatobiliary enzyme levels (Table 2). Contrast-enhanced computed tomography demonstrated an enhancing mass at the ampulla of Vater from the arterial to equilibrium phase, accompanied by dilatation of the bile duct and main pancreatic duct. Para-aortic lymphadenopathy and multiple hepatic lesions were also observed (Figure 2). EUS revealed a 25-mm heterogeneously hypoechoic mass at the ampulla of Vater, with slightly irregular margins. Contrast-enhanced EUS using perflubutane demonstrated hyperenhancement of the lesion (Figure 3).

**Table 2:** Laboratory findings on admission.

Laboratory parameter	Result	Unit	Reference range
Red blood cell count	4.03	×10^6^/µL	3.86-4.92
Hemoglobin	12.7	g/dL	11.6-14.8
Hematocrit	38.8	%	35.1-44.4
White blood cell count	5000	/µL	3,300-8,600
Platelet count	27.8	×10^4^/µL	15.8-34.8
Prothrombin time activity	79.4	%	70-130
Activated partial thromboplastin time	23.6	s	24-34
Total protein	7.3	g/dL	6.6-8.1
Albumin	3.9	g/dL	4.1-5.1
Total bilirubin	7.5	mg/dL	0.4-1.5
Direct bilirubin	5.6	mg/dL	≤0.4
Aspartate aminotransferase	84	U/L	13-30
Alanine aminotransferase	149	U/L	7-23
Alkaline phosphatase	198	U/L	38-113
Gamma-glutamyl transpeptidase	544	U/L	9-32
Lactate dehydrogenase	246	U/L	123-222
Blood urea nitrogen	22.3	mg/dL	8.0-20.0
Creatinine	1.58	mg/dL	0.46-0.79
Sodium	137	mEq/L	138-145
Potassium	4.1	mEq/L	3.6-4.8
Chloride	106	mEq/L	101-108
Carbohydrate antigen 19-9	35.9	U/mL	≤37
Carcinoembryonic antigen	32.6	ng/mL	≤5.0
C-reactive protein	1.32	mg/dL	≤0.14

Because no tumor exposure was observed on the duodenal mucosal surface, histological diagnosis by conventional forceps biopsy was considered difficult. Therefore, EUS-FNB was performed using a 22-gauge needle (Figure 3).

**Figure 4:**
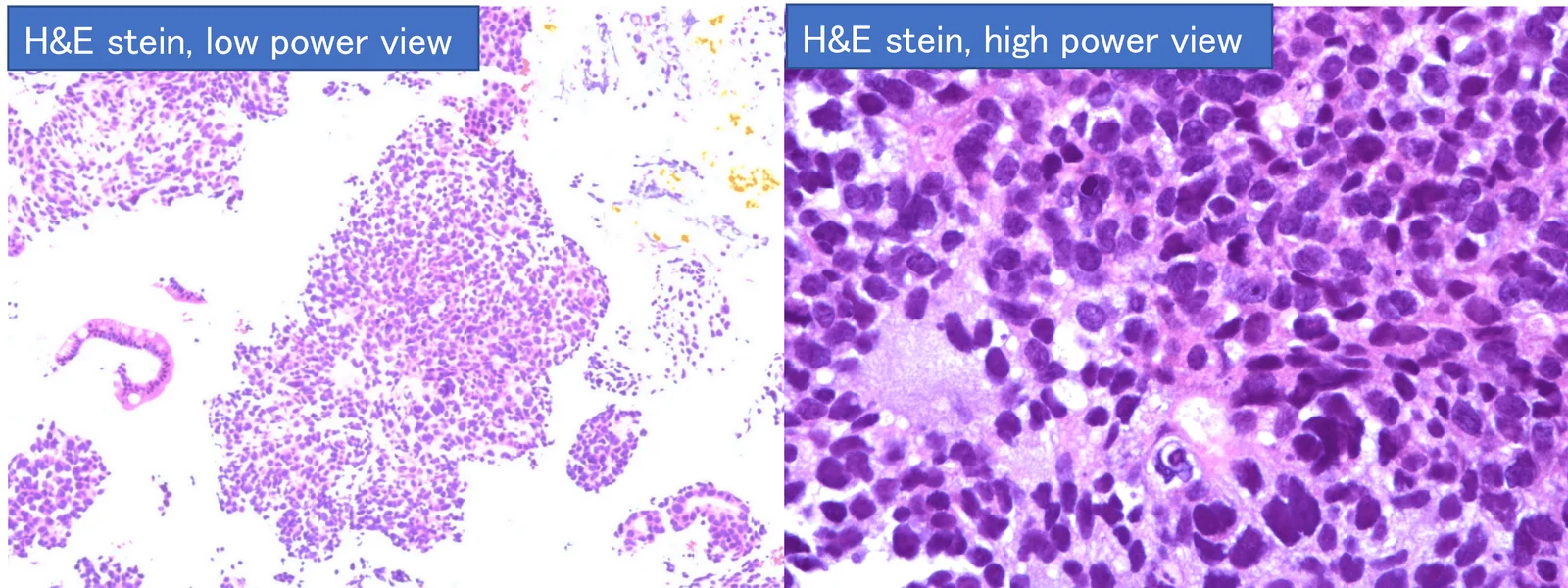
Histopathological findings: hematoxylin and eosin staining. Histopathological examination demonstrated solid nests of highly atypical small- to medium-sized tumor cells with focal rosette-like structures, irregular nuclei, scant cytoplasm, and a high nuclear-to-cytoplasmic ratio, consistent with small-cell neuroendocrine carcinoma.

**Figure 5:**
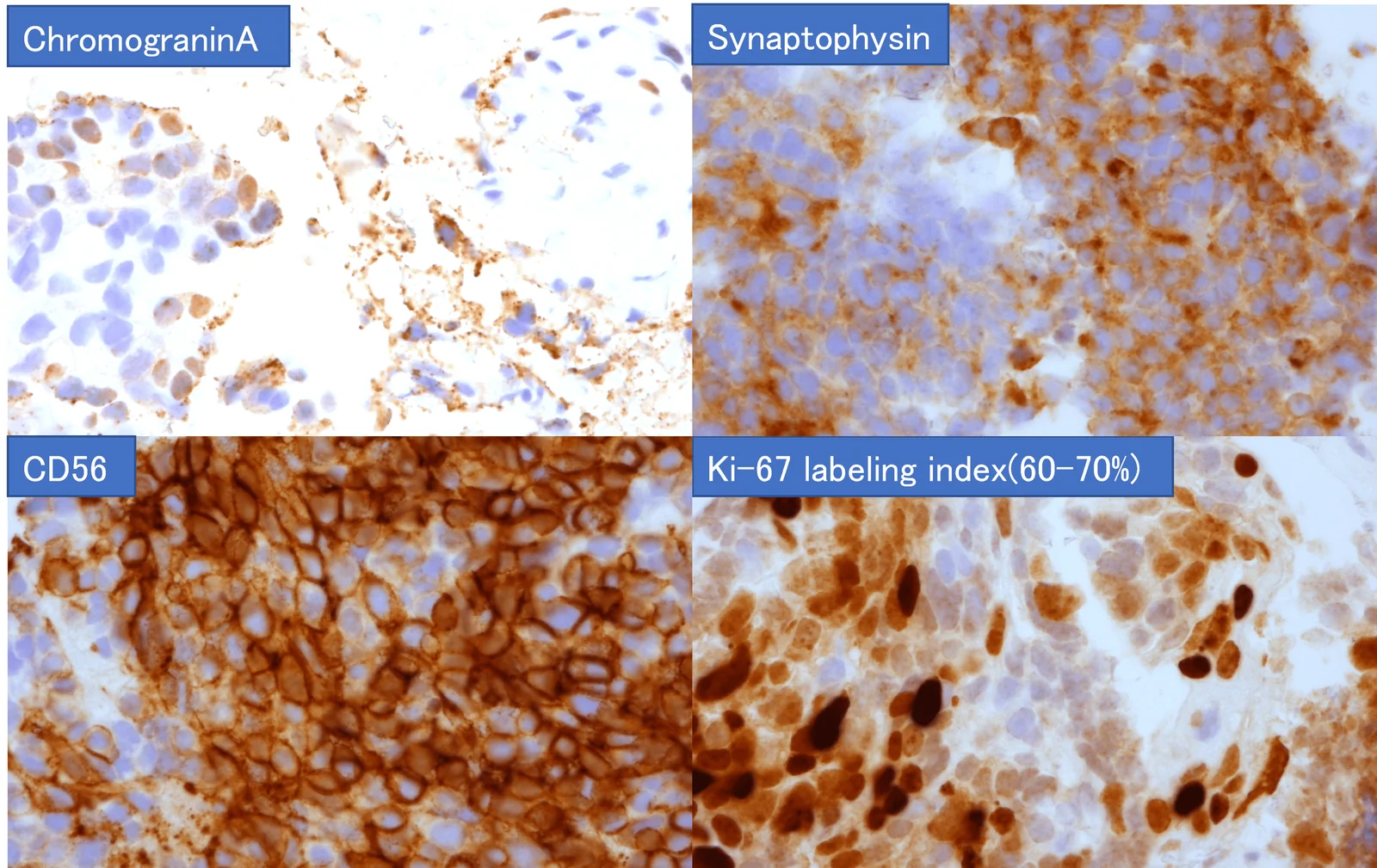
Histopathological images: immunohistochemical staining. Immunohistochemical staining demonstrated positivity for chromogranin A, synaptophysin, and CD56. The Ki-67 labeling index was 60%-70%. Additional immunohistochemical analyses demonstrated positivity for INSM1 and CDX2 and negativity for estrogen receptor and GATA3 (representative images not shown).

Histopathological examination demonstrated solid nests of carcinoma with focal rosette-like structures. The tumor consisted of small- to medium-sized cells with irregular nuclei, scant cytoplasm, and a high nuclear-to-cytoplasmic ratio, consistent with small-cell neuroendocrine carcinoma (Figure 4). The mitotic count was 5 mitoses per 10 high-power fields, and no definite tumor necrosis was identified. Immunohistochemical staining demonstrated positivity for chromogranin A, synaptophysin, CD56, INSM1, and CDX2, with a Ki-67 labeling index of 60%-70% (Figure 5). Estrogen receptor and GATA3 were negative, supporting a primary ampullary origin rather than metastatic breast carcinoma. Based on these morphological and immunohistochemical findings, the tumor was diagnosed as small-cell NEC of the ampulla of Vater.

The patient was diagnosed with small-cell NEC of the ampulla of Vater with synchronous liver and para-aortic lymph node metastases. Endoscopic retrograde biliary drainage was performed using a 7-Fr, 5-cm straight plastic stent, resulting in prompt resolution of obstructive jaundice. Chemotherapy with carboplatin and etoposide was subsequently initiated. However, computed tomography performed approximately two months after treatment initiation demonstrated progression of the liver metastases despite two treatment cycles. Because her underlying chronic kidney disease and chronic heart failure limited further treatment options, supportive care was initiated. The patient died approximately four months after diagnosis.

## Discussion

This case highlights the highly aggressive clinical course of ampullary NEC. The patient had undergone abdominal ultrasonography and magnetic resonance cholangiopancreatography two months earlier and esophagogastroduodenoscopy one year earlier, none of which demonstrated ampullary abnormalities, biliary dilatation, or metastatic disease. Nevertheless, she presented with a 25-mm ampullary tumor accompanied by synchronous liver and para-aortic lymph node metastases and died approximately four months after diagnosis, despite platinum-based chemotherapy. This clinical course is consistent with the highly aggressive biological behavior of poorly differentiated NECs, which are characterized by rapid progression, early distant metastasis, and poor survival [2].

Patients with branch-duct IPMNs undergo periodic surveillance to detect morphological progression and the development of pancreatic ductal adenocarcinoma. Previous studies have shown that pancreatic ductal adenocarcinoma may occasionally become clinically apparent during surveillance despite previously unremarkable imaging findings [6]. In the present case, however, the newly detected lesion was ampullary small-cell NEC rather than pancreatic ductal adenocarcinoma. To our knowledge, no direct association between IPMN and ampullary NEC has been established, and we therefore consider the coexistence of these two diseases to be incidental rather than causal.

Ampullary NEC is an uncommon but highly malignant subtype of ampullary neuroendocrine neoplasms. Previous multicenter studies have demonstrated that many patients present with lymph node or distant metastases and have an extremely poor prognosis despite multimodal treatment [3]. Compared with previously reported cases, our patient exhibited an exceptionally aggressive clinical course, developing widespread metastatic disease despite the absence of detectable ampullary abnormalities on surveillance examinations only a short time before diagnosis.

Histopathological diagnosis of ampullary NEC can be challenging, particularly when the lesion is not exposed on the duodenal mucosal surface. In the present case, conventional forceps biopsy was considered unlikely to provide a definitive diagnosis because no mucosal tumor exposure was observed. EUS-TA has been reported to improve the diagnostic yield for selected ampullary lesions in which conventional biopsy is difficult or inconclusive [5,7]. Although EUS-TA may occasionally be limited by small tissue samples or tissue fragmentation, adequate tissue was successfully obtained in the present case, allowing comprehensive histopathological and immunohistochemical evaluation, including determination of the Ki-67 labeling index. Furthermore, the diagnosis of a primary ampullary NEC was supported by diffuse neuroendocrine marker expression, together with CDX2 positivity and negative estrogen receptor and GATA3 staining, making metastatic breast carcinoma unlikely.

For localized gastroenteropancreatic NEC, surgical resection is recommended whenever feasible [8]. However, because most patients present with advanced metastatic disease, systemic platinum-based chemotherapy remains the standard first-line treatment despite generally poor outcomes [8,9]. In the NORDIC NEC study, advanced gastrointestinal NEC showed limited responsiveness to platinum-based chemotherapy and poor overall survival [9]. Similarly, despite prompt biliary drainage and carboplatin-etoposide chemotherapy, our patient experienced rapid disease progression within approximately two months, further illustrating the highly aggressive biological behavior of ampullary NEC.

Taken together, this case underscores two important clinical messages. First, newly developed morphological changes of the major papilla should be carefully evaluated even when recent surveillance studies have shown no abnormalities. Second, EUS-guided fine-needle biopsy may represent a valuable diagnostic option for non-exposed ampullary lesions in which conventional biopsy is unlikely to provide sufficient tissue for an accurate pathological diagnosis.

## Conclusions

We report a case of rapidly progressive NEC of the ampulla of Vater that became clinically apparent during IPMN surveillance. This case emphasizes the aggressive behavior and poor prognosis of ampullary NEC and the need for careful assessment of newly detected ampullary abnormalities. It also demonstrates the usefulness of EUS-FNB for establishing a pathological diagnosis in a non-exposed ampullary lesion.
